# Pharmacokinetic analysis of linezolid for multidrug resistant tuberculosis at a tertiary care centre in Mumbai, India

**DOI:** 10.3389/fphar.2022.1081123

**Published:** 2023-01-04

**Authors:** Juan Eduardo Resendiz-Galvan, Prerna R. Arora, Mahmoud Tareq Abdelwahab, Zarir F. Udwadia, Camilla Rodrigues, Amita Gupta, Paolo Denti, Tester F. Ashavaid, Jeffrey A. Tornheim

**Affiliations:** ^1^ Division of Clinical Pharmacology, Department of Medicine, University of Cape Town, Cape Town, South Africa; ^2^ Research Laboratories, P. D. Hinduja National Hospital and Medical Research Centre, Mumbai, India; ^3^ Division of Respiratory Medicine, P. D. Hinduja National Hospital and Medical Research Centre, Mumbai, India; ^4^ Center for Infectious Diseases in India, Division of Infectious Diseases, Johns Hopkins University School of Medicine, Baltimore, MD, United States; ^5^ Center for Tuberculosis Research, Division of Infectious Diseases, Johns Hopkins University School of Medicine, Baltimore, MD, United States; ^6^ Johns Hopkins Bloomberg School of Public Health, Johns Hopkins University, Baltimore, MD, United States

**Keywords:** MDR-TB (multidrug resistant-TB), linezolid (LZD), pharmacokinetics, NONMEM modelling, pharmacometrics

## Abstract

Linezolid is an oxazolidinone used to treat multidrug-resistant tuberculosis (MDR-TB), including in the recently-endorsed shorter 6-month treatment regimens. Due to its narrow therapeutic index, linezolid is often either dose-adjusted or discontinued due to intolerance or toxicity during treatment, and the optimal balance between linezolid efficacy and toxicity remains unclear. India carries a significant burden of MDR-TB cases in the world, but limited information on the pharmacokinetics of linezolid and minimum inhibitory concentration (MIC) distribution is available from Indian MDR-TB patients. We enrolled participants from a tertiary care centre in Mumbai, India, treated for MDR-TB and receiving linezolid daily doses of 600 or 300 mg. Pharmacokinetic visits were scheduled between 1 and 15 months after treatment initiation to undergo intensive or sparse blood sampling. Linezolid concentration *versus* time data were analysed using non-linear mixed-effects modelling, with simulations to evaluate doses for different scenarios. We enrolled 183 participants (121 females), with a median age of 26 years (interquartile range [IQR] 21–35), weight 55.0 kg (IQR 45.6–65.8), and fat-free mass 38.7 kg (IQR 32.7–46.0). Linezolid pharmacokinetics was best described by a one-compartment model with first-order elimination allometrically scaled by fat-free mass and transit compartment absorption. The typical clearance value was 3.81 L/h. Simulations predicted that treatment with 300 mg daily achieves a high probability of target attainment (PTA) when linezolid MIC was ≤0.25 mg/L (61.5% of participant samples tested), while 600 mg daily would be required if MIC were 0.5 mg/L (29% of samples). While linezolid 300 mg daily is predicted to achieve effective targets for the majority of adults with MDR-TB, it failed to achieve the therapeutic target for 21% participants. A dose of 600 mg had a PTA >90% for all susceptible samples, but with a higher likelihood of exceeding toxicity thresholds (31% vs 9.6%). These data suggest potential benefit to individualized dosing taking host and microbial characteristics into account to improve the likelihood of treatment efficacy while minimizing risk of toxicity from linezolid for the treatment of MDR-TB. Further prospective evaluation in different clinical settings is urgently needed to inform safety and efficacy of these lower doses.

## Introduction

Multidrug-resistant TB (MDR-TB), defined as tuberculosis caused by *Mycobacterium tuberculosis (Mtb)* isolates resistant to isoniazid and rifampicin, remains a major public health threat. According to the World Health Organization (WHO), India faces one of the highest global burdens of both TB (26%) (World Health Organization, 2020a) and MDR-TB (27%). (World Health Organization, 2020a). Linezolid, an oxazolidinone with demonstrated activity against *Mtb,* is recommended as one of the primary drugs for treatment of MDR-TB in shorter and longer regimens. (Schecter et al., 2010; World Health Organization, 2020b).

Linezolid has a rapid and extensive absorption after oral administration with high bioavailability (∼100%). (Qin et al., 2022). Approximately 30% is excreted unchanged through the kidneys, with non-renal clearance accounting for 65% of total clearance, resulting in a terminal half-life of 3.5–7 h. (Macgowan, 2003; Stalker and Jungbluth, 2003). Linezolid works by binding the *Mtb* 23S ribosomal subunit and preventing protein synthesis, (Livermore, 2003), but, unfortunately, it also affects the human mitochondrial 16 S rRNA subunit leading to mitochondrial toxicity, causing dose reductions or interruptions of intended linezolid treatment course. (Wasserman et al., 2016; Conradie et al., 2020). The significant toxicity profile and ongoing uncertainty about optimal linezolid dosing for MDR-TB confounds clinical efforts to balance efficacy, resistance suppression, and adverse events. (Wasserman et al., 2016). Previous studies have proposed a minimum concentration (C_min_) threshold of 2 mg/L as a marker of adverse effects related to mitochondrial toxicity from linezolid. (Song et al., 2015).

The most widely-used linezolid dose for MDR-TB supported by trial evidence is 600 mg daily, (Conradie et al., 2022), but this frequently is reduced to 300 mg daily because of toxicity, which may fail to achieve the therapeutic target for *Mtb*. (Maartens and Benson, 2015). Previous studies suggest that efficacy is driven by the ratio of the area under the curve of unbound linezolid divided by minimal inhibitory concentration (ƒAUC/MIC), with a target ratio of >100 likely reach an appropriate exposure and minimize toxicity. (Bolhuis et al., 2018). Population-specific pharmacokinetic (PK) models are paramount to ensuring that patients receive the best-possible dosing to achieve the efficacy target. Previous population PK studies of linezolid for MDR-TB have been performed in patients in South Africa, (Garcia-Prats et al., 2019; Abdelwahab et al., 2021; Padmapriyadarsini et al., 2022), Italy, (Tietjen et al., 2022), Brazil, (Alghamdi et al., 2020), United States, (Alghamdi et al., 2020), and China, (Zhou et al., 2022), but despite the global burden, there are limited population PK data from India. Given possible differences in comorbidities, coadministered drugs, diet, and other unmeasured characteristics, we sought to better understand the PK of linezolid in an Indian population. Our study developed a population PK model using linezolid blood levels collected through a cohort study of Indian adolescents and adults with MDR-TB and explored the probability of target attainment (PTA) with daily linezolid treatment at doses of 600 and 300 mg.

## Material and methods

### Study population

Data were collected through a prospective observational study of adolescents and adults treated for MDR-TB at a tertiary care centre in Mumbai, India that has been described elsewhere. (Udwadia et al., 2019; Tornheim et al., 2020; Tornheim et al., 2022). Briefly, treatment-naïve individuals were enrolled from October 2015-January 2022 at the start of drug susceptibility testing-based personalized 24-month treatment regimens, informed by WHO and national guidelines at a private sector hospital. (World Health Organization, 2020b; Ministry of Health & Family Welfare Government of India, 2021). Participants were followed longitudinally with clinical characteristics, laboratory and imaging results, side effects, and treatment outcomes recorded throughout participation. Adult participants provided written informed consent prior to enrolment and 15–18-year-old participants provided written informed assent with written informed consent for participation provided by their legal guardians. For participants treated with linezolid, an initial dose of 600 mg daily was prescribed, but was reduced to 300 mg daily for those with linezolid-associated toxicity (either peripheral neuropathy, anaemia with haemoglobin <10 g/dl, thrombocytopenia <100,000/µL, or leukopenia with <1,000 neutrophils/µl). Participants at higher risk of toxicity due to malnutrition, underweight, or neuropathy-associated comorbidities like diabetes and alcohol use were started at lower doses of 300 mg daily. This study was approved by the institutional review boards at the P.D. Hinduja National Hospital and Medical Research Centre (“Hinduja Hospital”, IRB00012235) and the Johns Hopkins University School of Medicine (IRB00076738, IRB00012235).

### Data collection

Study participants provided blood samples for PK analysis at 1, 2, 6, and 12 months after the treatment initiation, with blood samples collected before and 2 h after observed daily linezolid doses. A random subset of participants provided additional consent for collection of intensive PK sampling at the first or second month time points, with blood collection before and 1, 2, 4, 6, and 8 h after observed daily linezolid doses. Due to the COVID-19 pandemic and associated rescheduling of participant visits, sparse sampling was accepted through the 15th month of MDR-TB treatment and intensive sampling was accepted through the fourth month of MDR-TB treatment.

Isolates collected from participants with culture-positive MDR-TB were submitted for minimum inhibitory concentration (MIC) testing using custom Sensititre plates manufactured by ThermoFisher. Isolates were cultured on Löwenstein–Jensen media and evaluated weekly by laboratory staff until late-log phase growth, at which time they were agitated in saline tween before inoculation into Sensititre plates by a Thermo Scientific AIM autoinoculator. Isolates were tested at linezolid concentrations of 0.12, 0.25, 0.5, 1, 2, 4, and 8 mg/L, which were quality-controlled using two drug-free control wells on each plate and parallel testing of the H37Rv laboratory strain on an identical plate with each batch to confirm expected results. Plates were read using a mirror box on 10-, 14-, 21-, and 28-day following inoculation by two independent readers to ensure concordance, with the final value selected as the first date with adequate growth in both wells. When the two readers reported discordant values, a third independent reader adjudicated the final result.

Linezolid concentrations were measured at the Hinduja Hospital laboratory. Blood samples were collected without anticoagulants and centrifugated at 3,000 rpm for 10 min. Serum was then collected, aliquoted, and stored at -80°C until analysis, depending on laboratory schedule. Quantification employed a commercially available enzyme immunoassay (ARK™ Linezolid Assay) according to manufacturer’s instructions with a calibration range of 0.75–30 mg/L, limit of quantification (LOQ) of 0.75 mg/L, limit of detection (LOD) of 0.071 mg/L, and tri-level controls. Samples with concentrations >30 mg/L were assayed by dilution with the corresponding zero calibrator and estimated using the dilution factor.

### Model building and analysis

Linezolid serum concentration-versus-time data were analysed using the non-linear mixed-effect model software NONMEM v7.4 (ICON Development Solutions, Hanover, MD, United States) (Beal et al., 2013) and the algorithm first-order conditional estimation with ε-η interaction (FOCE INTER). Graphical diagnostics, managing and organization of the models were handled using Perl-speaks-NONMEM, Xpose4 (Jonsson and Karlsson, 1998) embedded in R, and Pirana (Certara, Princeton, NJ, United States), (Keizer et al., 2013), respectively.

The overall strategy for model development started with only intensively sampled data. Once the model satisfactorily described the intensively sampled data, the sparse sampling data were added for parameter reassessment and additional covariate testing using the complete dataset. One- and two-compartments disposition models, linear and non-linear elimination kinetics, first-order absorption (with and without lag), and transit compartments (Savic et al., 2007) were investigated to determine the best structural model. Random effects were included as between-subject variability (BSV) on all disposition parameters and between-occasion variability (BOV) (Karlsson and Sheiner, 1993) for absorption parameters, assuming a lognormal distribution. Non-observed doses and pre-dose concentrations were considered independent occasions from observed doses (during sample collection) and subsequent concentrations. Residual unexplained variability (RUV) was modelled testing both additive and proportional components, with the additive component bound to ≥20% of the LOQ. All concentration values above the LOD, including those below the LOQ (BLQ), were included in the model as actual values measured, similar to the published “all data” method. (Keizer et al., 2015). Concentrations below the LOD (BLD) were censored by the laboratory, and incorporated in the model by adapting the M6 method. (Beal, 2001). Briefly, BLD concentrations were imputed as LOD/2 (0.0355 mg/L) and the additive component of RUV was inflated by LOD/2 to acknowledge the extra uncertainty and compensate for the effect of imputation. If a series of consecutive BLD concentrations was present, only one value was included in the model, the last one if in the absorption phase and the first one in the elimination phase, while additional BLD concentrations were excluded for parameter estimation and retained for simulation-based diagnostics.

Allometric scaling was evaluated on disposition parameters using total body weight and fat-free mass (FFM). (Anderson and Holford, 2008). Continuous covariates included in model assessments were age, serum creatinine, Cockcroft-Gault creatinine clearance estimates, (Cockcroft and Gault, 1976), and days on linezolid treatment. Categorical covariates assessed included participant sex and additional drugs prescribed. Differences in covariate distributions between participants with intensive and sparse data were evaluated by the Fisher’s exact and Wilcoxon signed rank tests for categorical and continuous variables, respectively, with *p* < 0.05 considered to indicate between-group differences. Covariate relationships were screened and evaluated based on physiological plausibility and improvement in model diagnostics (including goodness of fit plots and visual predictive checks) using a stepwise approach. Forward inclusion of covariates required NONMEM objective function value (ΔOFV) reduction of ≥3.84 for inclusion of one degree of freedom (*p* < 0.05), followed by backward elimination with a ΔOFV≥6.63 (*p* < 0.01) for retention of one degree of freedom. Uncertainty in final model parameter estimates was quantified using the sampling importance resampling method. (Dosne et al., 2017).

### Simulations

Monte Carlo simulations were performed using final model parameter estimates to evaluate the PTA defined as the percentage of simulated individuals above the exposure targets. A minimum concentration (C_min_) > 2 mg/L was employed as the threshold for increased risk of mitochondrial and haematological toxicity. (Song et al., 2015). The pharmacokinetic-pharmacodynamic (PK-PD) index for efficacy to estimate the PTA was ƒAUC_0-24_/MIC≥100. (Bolhuis et al., 2018). The ƒAUC was estimated considering an unbound fraction of 70%. (Qin et al., 2022). Exposure was estimated with doses of 300 and 600 mg once daily using an *in silico* population created by repetition of demographic characteristics from participants with drug-susceptible or MDR-TB from previous studies. (Diacon et al., 2007; Wilkins et al., 2008; Pepper et al., 2010; Chigutsa et al., 2011; McIlleron et al., 2012; Smythe, 2016; Chirehwa et al., 2019).

## Results

### Demographics and clinical profile

Data were available for 183 participants, 121 of whom were female, with median age of 26 years (interquartile range, IQR: 21–35), weight 55 kg (IQR: 45–66), and FFM 39 kg (IQR: 33–46), respectively. Five participants were HIV positive. In addition to linezolid, susceptibility-guided multidrug MDR-TB treatment regimens taken at the time of PK sampling. These included coadministration of moxifloxacin (91% of participants), cycloserine (86%), clofazimine (81%), pyrazinamide (44%), ethambutol (39%), kanamycin (27%), para-aminosalicylic acid (27%), bedaquiline (25%), and ethionamide (23%). Table 1 shows the study population’s baseline characteristics, which were not significantly different between participants with intensive and sparse sampling data.

**TABLE 1:** Characteristics of the study population.

Variable (units)	Intensive sampling n = 48	Sparse sampling n = 182
Age (years)	26 (21–32)	26 (21–35)
Females (n)	32 (66.7)	121 (66.5)
Weight (kg)	56.0 (46.0–66.1)	54.8 (45.4–65.8)
Height (m)	1.58 (1.51–1.66)	1.59 (1.53–1.68)
Fat-free mass (kg)	39.5 (33.1–46.5)	38.5 (32.7–45.8)
Serum creatinine (mg/L)	0.7 (0.6–0.8)	0.70 (0.6–0.8)
HIV positive (n)	2 (4.2)	3 (1.6)

^a^ Data presented as median (interquartile range) or number (percent).

No significant differences were identified between the two sampling groups for categorical or continuous covariates evaluated with the Fisher’s exact test (Sprent 2011) and the Wilcoxon signed rank test, respectively.

**FIGURE 1:**
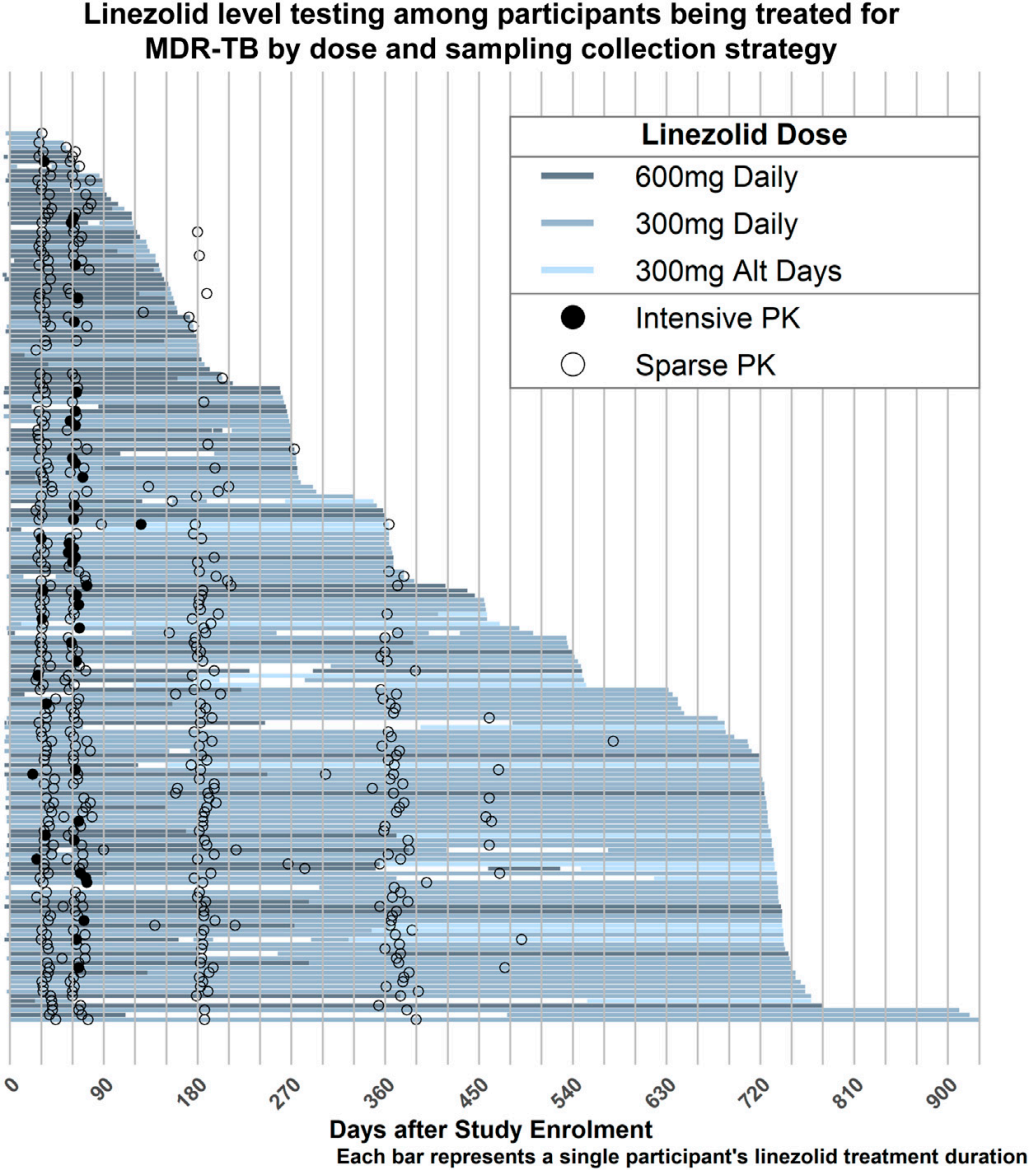
Duration of linezolid treatment. Each horizontal line represents a single participant, with the colour coding denoting the dose of linezolid prescribed over that time period. Most participants were treated for 24-month, though dose adjustments frequently occurred due to treatment-associated toxicity. Filled circles indicate intensive sampling visits and open circles indicate sparse sampling visits.

Intensive and sparse blood sampling was performed in 48 participants from 1 to 4 months and from 1 to 15 months after treatment initiation, respectively. Intensive sampling was most frequently performed at 2 months (36 participants, 75%), followed by 1 month (9 participants, 19%) after treatment initiation. This comprised a total of 1,181 linezolid measurements, 288 from intensive and 893 from sparse sampling, and included 74 BLQ and 123 BLD values. Treatment duration and the schedule of the PK visits for each participant is demonstrated in Figure 1. Most participants were initially prescribed 600 mg daily, but a large proportion (75%) were dose-reduced to 300 mg daily due to toxicity by the time of intensive PK (Figure 1). This represents a median time to dose reduction of 69 days (IQR: 63–74). Most PK visits assessed doses of 300 mg (89, 50.3%) or 600 mg daily (86, 48.6%), while a minority (2 visits) assessed linezolid at 300 mg dosed every other day. Of 166 cultured isolates, 11 (6.5%) demonstrated MICs above the critical concentration of 1 mg/L. Among susceptible isolates, we found five to have an MIC of 1 mg/L (3% of all samples), 48 to have an MIC of 0.5 mg/L (29%), 61 to have an MIC of 0.25 mg/L (36.5%), and 41 to have MICs <0.25 mg/L (25%).

### Population pharmacokinetic model

**FIGURE 2:**
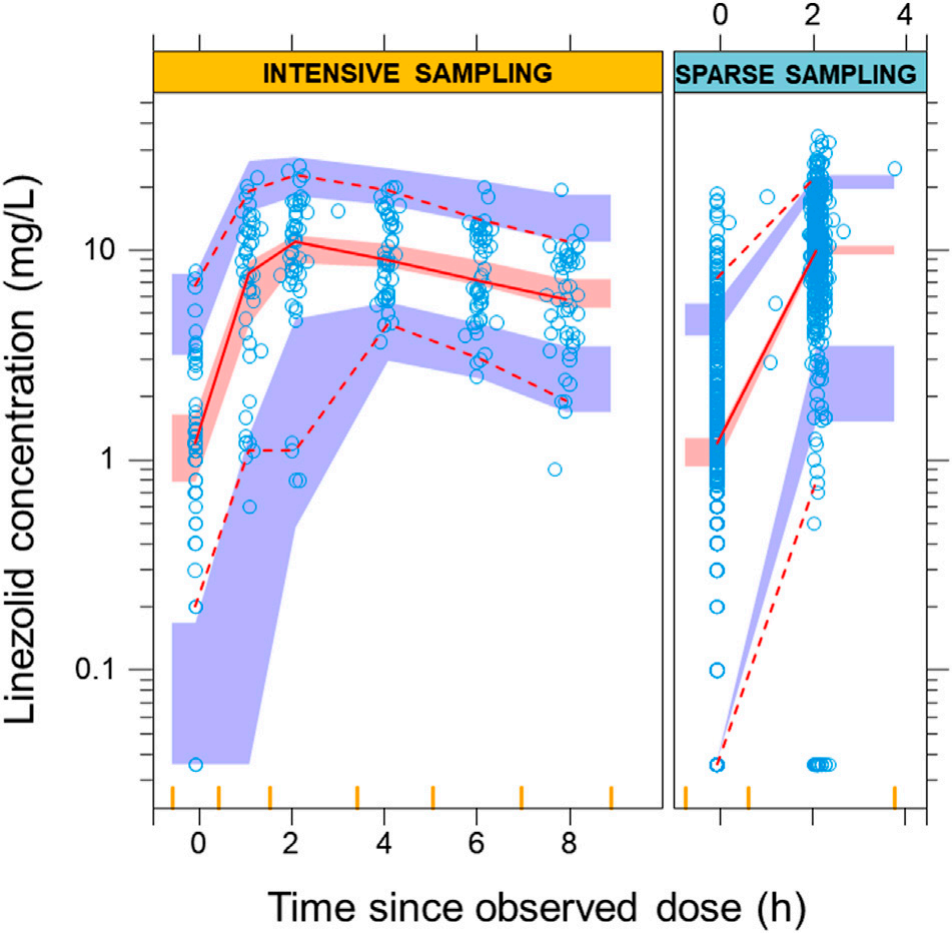
Visual predictive check from the final model. The solid and dashed lines correspond to the fifth, 50th, and 95th percentiles from the original observations (blue circles), while the shaded areas represent the 95% confidence intervals for the same percentiles of the model predictions. The left panel shows the intensive data with sampling points from pre-dose up to 8 h post-dose. The right panel shows the predictions for the sparse observations at pre-dose and 2 h post-dose The bins for sampling points are showed as vertical yellow lines on the *x*-axis.

Linezolid PK was best described by a one-compartment disposition model with first-order elimination and first-order absorption including a chain of transit compartments. The final PK parameters are presented in Table 2 and a visual predictive check stratified by type of data (intensive and sparse sampling) in Figure 2, showing an adequate interpretation of the observations by the final model. Allometric scaling using FFM best described the effect of body size (ΔOFV 15.6) on the disposition parameters and provided a better fit than using total body weight (ΔOFV 8.57). The model estimated the typical PK parameters for a participant with FFM 39.5 kg; clearance was 3.81 L/h and volume of distribution 31.2 L. Creatinine clearance, serum creatinine, age, and coadministration of linezolid with other TB drugs did not show statistically significant associations with model-derived PK parameters. Pre-dose concentrations were observed to be more variable and more poorly predicted than concentrations measured after observed doses administered at the clinic. To account for this larger variability, we tested the inclusion of a factor increasing the between-occasion variability for all absorption parameters following unobserved doses taken at home. This significantly improved the model fit (ΔOFV = 36.7, 1 degree of freedom, *p* < 0.001).

**TABLE 2:** Final population pharmacokinetic parameters.

Parameter	Typical value (95% CI^a^)	Variability^b^ (95% CI^a^)
Clearance (L/h)^c^	3.81 (3.41—4.25)	BSV: 31.9 (23.4—42.4)
Volume of distribution (L)^c^	31.2 (29.3—33.4)	
Absorption rate constant (1/h)	2.31 (1.89—4.32)	BOV: 104 (82.0—108)
Mean transit time (h)	0.666 (0.481—0.884)	BOV: 80.5 (62.7—105)
Number of transit compartments (n)	20.5 (12.7—34.9)	
Bioavailability	1 FIXED	BOV: 18.4 (10.1—24.4)
Variability factor for unobserved doses^d^ (-fold change)	1.86 (1.44—1.15)	
Proportional error (%)	5.24 (3.54—6.97)	
Additive error (mg/L)	0.362 (0.271—0.473)	

BSV, Between-subject variability; BOV, Between-occasion variability.

^a^ Sampling importance resampling (SIR) was used to obtain the 95% Confidence interval (CI).

^b^ The unobserved dose factor was considered for the administration of unobserved doses (e.g., doses taken at home on days prior to blood sampling) and consequently the impact on the pre-dose concentration quantified with an extra parameter. This extra variability accounts for absorption rate constant, mean transit time, and bioavailability.

^c^ Allometric scaling was used to estimate clearance and volume of distribution for the typical patient with a fat-free mass of 39.5 kg.

^d^ BSV, and BOV, were assumed to be log-normally distributed and reported as approximate (%CV).

### Probability of target attainment

**FIGURE 3:**
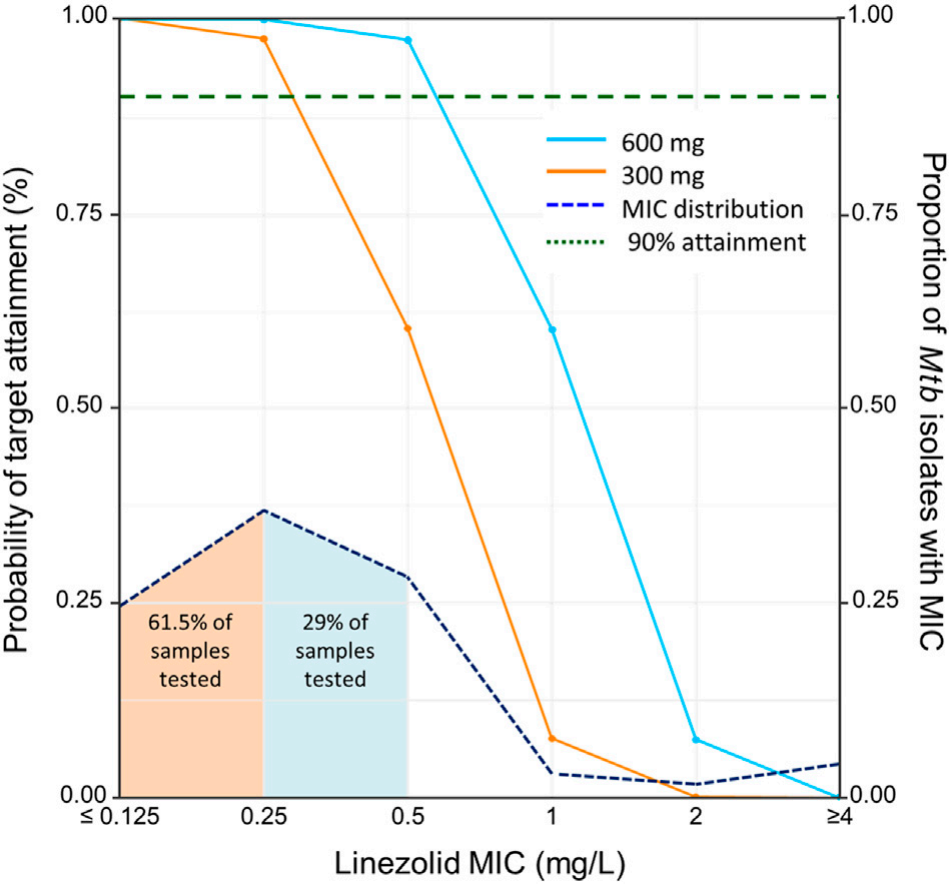
Probability of target attainment by dose and minimum inhibitory concentration. Solid lines indicate the probability of target attainment (PTA) on the primary *y*-axis (on left) based on area under the curve of unbound linezolid divided by MIC (ƒAUC/MIC), adjusting for the linezolid minimum inhibitory concentration (MIC) on the *X*-axis. Lines indicate simulated doses of 600 mg once daily (blue solid line) and 300 mg once daily (orange solid line). The horizontal dashed green line indicates the 90% of attainment when ƒAUC/MIC ≥100. The dot-dashed line indicates the distribution of linezolid MIC of *Mycobacterium tuberculosis* isolates cultured from 166 study participants, with the proportion of all tested isolates with each MIC indicated on the secondary *y*-axis (on right). The shaded area under the dot-dashed line indicates the proportion of tested samples with the corresponding MIC (color indicates that of the lowest dose with PTA >90% for that MIC).

Considering a target of ƒAUC_0-24_/MIC ≥100, the PTA for a linezolid dose of 600 mg once daily was >99% against samples with linezolid MICs ≤0.25 mg/L (61.5% of isolates tested in this study), 97% for MIC = 0.5 mg/L (29% of isolates), 60% when MIC is 1 mg/L (3% of isolates), and <8% when MIC is > 1 mg/L (6.5% of isolates). The PTA for linezolid at a dose of 300 mg once daily was >97% for samples with linezolid MICs ≤0.25 mg/L, but only 60% for samples with MIC of 0.5 mg/L, 8% for samples with MIC of 1 mg/L, and <1% for samples with MIC >1 mg/L (Figure 3). When our model was applied to the total population of study participants with the MIC results for their own isolates, this corresponds to probabilities of 90% and 79% that participants would achieve their expected target at doses of 600 and 300 mg daily, respectively. Regarding the toxicity thresholds, 31% and 9.6% of the simulated patients exceeded a C_min_ of 2 mg/L with 600 and 300 mg, respectively.

## Discussion

In this study, we describe the population PK of linezolid in a cohort of Indian adults and adolescents treated for MDR-TB and evaluate dosing strategies to balance treatment efficacy and toxicity. We found that while 300 mg daily is expected to be effective against strains with MICs 0.25 mg/L or lower, 600 mg daily may be more appropriate for MIC levels of 0.5–1 mg/L, albeit with a higher likelihood of treatment-associated toxicity.

Our PK model identified one-compartment disposition with first-order elimination, and first-order absorption with transit compartments. This is consistent with previous studies on linezolid PK, which have primarily employed similar one-compartment models for both drug-susceptible TB and MDR-TB. (Alghamdi et al., 2020; Abdelwahab et al., 2021; Tietjen et al., 2022). Other studies have reported two-compartment disposition with Michaelis-Menten elimination when linezolid is dosed at 1,200 mg daily, (Imperial et al., 2022), or linezolid inhibiting its own clearance with repeated administration. (Plock et al., 2007; Mockeliunas et al., 2022). In our data, testing two-compartments disposition and saturation did not improve model fit, possibly due to differences in the sampling schedule and the fact that non-linear kinetics may not be evident at the lower doses prescribed to participants in our study. Due to the limitations of our overall study design, it was not possible to meaningfully evaluate autoinhibition, since that would require PK data from the first days of linezolid administration.

Interestingly, reports for linezolid PK in different populations show different values of clearance. The typical clearance estimated in our population, 3.81 L/h, is similar to the clearance of 3.57 l/h reported in South African patients from two separate clinical trials. (Abdelwahab et al., 2021). Higher clearance has been reported in other publications. For example, clearance was found to be 6.06 l/h in study participants from Brazil and the USA, (Alghamdi et al., 2020), 7.69 l/h in an Italian population, (Tietjen et al., 2022), and 4.59 l/h in a Chinese population. (Zhou et al., 2022). While part of the difference could be ascribed to the larger weight of the participants in the other cohorts, who were slightly heavier than those in our study and in South African studies, the difference persisted despite allometric scaling in our model. Other differences between studies that could have accounted for the variability in the parameters are the linezolid dose ranging from 300 to 600 mg once or twice daily, and the prolonged time on treatment for participants in this study. However, we did not find a significant effect of these covariates in the final model. New evidence has shown that linezolid is mainly metabolized to its inactive metabolites in the liver through oxidation of the morpholine ring by distinct isoforms CYP2J2, CYP4F2, and CYP1B1 of the Cytochrome P450 enzyme family. (Obach, 2022). This could also contribute to differences in linezolid metabolism based on differences in allele distribution frequencies between distinct populations. However, the presence of polymorphisms on these enzymes and their influence on linezolid metabolism needs to be evaluated in future studies. Importantly, we found high between-subject variability for clearance and between-occasion variability for absorption process within the Indian population studied in this cohort, suggesting a potential role for therapeutic drug monitoring to optimize linezolid treatment, though PK laboratory capacity is limited, particularly in high incidence settings.

According to our simulations, 300 mg daily would achieve a ƒAUC/MIC ≥100 in at least 97% of the simulated individuals when the MIC is ≤ 0.25 mg/L, while a dose of 600 mg daily would be more suitable for isolates with higher MICs. This dosing suggestion is similar to the one published for the South African cohort participants, (Abdelwahab et al., 2021), while higher doses have been proposed in studies finding higher clearance values. (Alghamdi et al., 2020; Mockeliunas et al., 2022). It is also important to highlight that the MIC distribution may vary between countries, and indeed we report lower MICs in this study than reported in South Africa. (Abdelwahab et al., 2021). From a toxicity perspective, our simulations suggest that 31% of people receiving 600 mg daily would exceed the literature-derived C_min_ threshold of 2 mg/L, (Song et al., 2015), compared to 9.6% of those receiving 300 mg daily While more data are needed to confirm the predictive value of this threshold for clinical application, particularly as competing toxicity thresholds are considered, (Wasserman et al., 2019a), the high proportion of people affected at either dose and the large between-subject variability we observed offers an argument in support of individualized therapy that considers clinical features such as fat-free mass and the extent of microbiological resistance to better identify the 31% at risk of toxicity as well as to ensure efficacy among those given lower or less frequent doses.

As TB control programs worldwide move to adopt linezolid-based treatments for MDR-TB, (Padmapriyadarsini et al., 2022; World Health Organization, 2022), it will be important to recognize that despite evidence of resistance in high-burden settings, (Tornheim et al., 2020), the majority of such treatment is prescribed in the absence of susceptibility testing for linezolid, let alone MIC testing. While infrequently discussed in the literature and rarely tested in clinical practice, linezolid resistance is identified with increasing frequency in high-burden settings where testing is performed. Previously considered to be rare, (Bharadwaj, 2021), resistance was noted to affect 1% of isolates tested in Mumbai, India in 2017, (Tornheim et al., 2017), with more recent publications documenting resistance among 6.7%, (Tornheim et al., 2020), similar to rates documented in China (6.9%). (Du et al., 2021). Among South African patients with MDR-TB and treatment failure, linezolid resistance has been reported among 33% of isolates. (Wasserman et al., 2019b). Given the increasing prescription of linezolid in shorter regimens with bedaquiline and pretomanid, with or without moxifloxacin, (World Health Organization, 2022), increased vigilance for emerging linezolid resistance is crucial to secure the success of TB elimination programs. (Bharadwaj, 2021).

Because the efficacy target for linezolid is normalized to MIC, improving local knowledge of drug resistance and MICs can help TB providers determine the MIC distributions to target with different linezolid dosing strategies, with or without assistance from PK models such as this one. Given the WHO-endorsed critical concentration of 1 mg/L, (World Health Organization, 2018), in the absence of local MIC knowledge, a dose of 600 mg would ensure a PTA ≥97% against MICs ≤0.5 mg/L, and which represented 90.5% of isolates in this study. This finding supports the results of the recently published ZeNIX trial demonstrating improved efficacy and toxicity of a 600 mg daily dose compared to other dosing strategies. (Conradie et al., 2022). While the use of higher doses such as 1,200 mg daily in the NIX-TB study may increase drug exposure, (Conradie et al., 2020), the high rates of toxicity-associated linezolid dose reduction or treatment interruption during 6 months of therapy (85%) leaves much room for improvement. Similarly, a dose of 600 mg twice-daily achieves a target attainment of 100% in simulation studies, but with >99% of the simulated individuals exceeding the safety levels. (Millard et al., 2018).

Our study had several limitations. This relatively small, single-site study may not be generalizable to non-Indian populations, populations with different rates of comorbid diseases such as HIV, diabetes, or malnutrition that affect absorption, children, or those with drug-susceptible tuberculosis with different concomitant treatments. Due to the small number of participants coinfected with HIV, we could not assess drug-drug interactions with antiretroviral therapy, which is an important factor that may influence the treatment in people living with HIV. Given that the majority of MDR-TB globally is not associated with HIV, however, our data are relevant to a large proportion of global cases. Additionally, model development was better informed by intensive than sparse PK data, which is easier to obtain in clinical settings, but is simultaneously less-informative because parameters estimation relies on a reduced number of observations within a dosing interval. For this reason, model building and assessment of PK parameters relied in an initial stage only on the intensive data, which was available for only 26% of participants. Additionally, the pre-dose concentrations from both intensive and sparse data were affected by larger variability than post-dose concentrations, likely due to self-reported dosing history. We tried to mitigate the effect of this uncertain information by allowing larger between-occasion variability for absorption parameters and bioavailability in the final model. As shown in the final visual predictive check, the model prioritized intensive data, but still described sparse data adequately. Finally, this non-interventional cohort study assessed the extent to which participants achieved literature-derived efficacy and toxicity targets. Future studies will need to evaluate the impact of model-derived treatment decisions on improved treatment outcomes and frequency of treatment-associated side effects.

## Conclusion

To the best of our knowledge, this is the first PK model for linezolid developed in a population of Indian patients with MDR-TB. We report values of clearance similar to those reported in South African patients. These results suggest that while linezolid dosed at 300 mg daily may be effective against isolates with MICs ≤0.25 mg/L, a dose of 600 mg is more likely to achieve the efficacy target for isolates with higher or unknown MICs and improved the PTA from 60% to 97% against such isolates, representing nearly a third of samples tested in this study. The high variability in multiple important PK parameters demonstrates a role for model-based, individualized therapy to optimize linezolid exposure. Additional prospective studies are needed to confirm these findings and evaluate their role in improving both efficacy and toxicity thresholds in clinical settings.

## Data Availability

The raw data supporting the conclusion of this article will be made available by the authors, without undue reservation.
